# Editorial: Innate immune responses in HIV controllers

**DOI:** 10.3389/fimmu.2023.1159278

**Published:** 2023-02-15

**Authors:** Christina Thobakgale, Stephanie Jost

**Affiliations:** ^1^ Faculty of Health Sciences, School of Pathology, University of the Witwatersrand, Johannesburg, South Africa; ^2^ Centre for HIV and STIs, National Institute for Communicable Diseases, Johannesburg, South Africa; ^3^ Division of Innate and Comparative Immunology, Center for Human Systems Immunology, Department of Surgery, Duke University School of Medicine, Durham, NC, United States

**Keywords:** HIV, elite controllers, innate immune responses, NK cells, dendritic cells

HIV-1 controllers comprise a heterogeneous group of people living with HIV-1 (PLWH) with the ability to maintain low viral loads and high CD4+ T cell counts for years in the absence of antiretroviral therapy (ART). Within these is a special group of individuals termed “elite controllers” (ECs) who durably maintain undetectable plasma HIV-1 RNA levels without medication. Immune profiles in these rare individuals have been proposed as a model to guide the development of a functional HIV-1 cure. Several protective immune signatures have been linked to better HIV-1 control but host factors described thus far only partly explain effective HIV-1 control (1, 2). In particular, a potent innate immune response is likely critical to contain viral replication early following infection and to promote spontaneous HIV-1 control. Nevertheless, the delineation of innate immune responses in HIV-1 controllers remains an understudied research area. This Research Topic collected contributions advancing progress made on understanding innate immunological mechanisms of spontaneous HIV-1 control and discusses gaps of knowledge and strategies to harness innate immune cells in future immunotherapeutics.

Among other roles, innate immune cells coordinate adaptive immune responses and accumulating evidence highlighting that both innate and adaptive arms of the immune response may be responsible for spontaneous control of HIV-1 is beginning to emerge (3, 4). For instance, in recent reports, myeloid dendritic cells (mDCs) from ECs exhibited increased abilities to sense HIV-1 through cytosolic pathways, consequently enhancing activation of CD8+T cells with a CD64+PD-L1_hi_ phenotype (5, 6). In the Original Research article by Martin-Gayo et al. the authors identify molecular circuits RNA-sensor RIG-I together with cGAS as key players in mediating the detection of HIV-1 in mDC from ECs. Their findings represent therapeutically actionable pathways for enhancing innate immune responses against HIV-1.

Submissions in this Research Topic include four excellent reviews that delve into some of the innate immune mechanisms linked to natural control of HIV-1, starting with a review from Shi et al. which presents an overview of innate immunity as the first line of defense against pathogen invasion and a bridge to induce adaptive immunity. The involvement of innate cells such as dendritic cells, natural killer (NK) cells, macrophages and natural killer T cells, the secretion of soluble cytokines and signaling pathways are emphasized and deemed critical to the natural control of HIV-1 infection. The involvement of both arms of the immune response described above can be demonstrated by the contribution of DCs to the enhancement of the overall HIV-1 specific T cell immunity observed in ECs (5). Furthermore, higher frequencies of NK cells and CD4+ T cells with antiviral activity were reported in HIV-1 controllers (7). Thus, innate molecules may down-regulate viral replication whilst reducing tissue damage and the seeding of the viral reservoir before the adaptive immune response kicks in.

NK cells are classically considered as effector cells of the innate immune system that promptly respond to HIV exposure and a plethora of studies have provided compelling evidence for their significant contribution to the immune control of HIV-1 (8, 9). While subsets of NK cells with potent anti-viral activity may confer protection, prolonged recruitment of highly activated NK cells may also contribute to immunopathology. The mini review article by Mensching et al. summarizes multiple NK cell functions that contribute to HIV-1 control and the disparate roles played by myeloid cells in antigen presentation, inflammation and HIV-1 persistence, and specifically addresses the importance of the crosstalk between blood monocytes or tissue macrophages and NK cells. The authors highlight several aspects of these interactions that could be either beneficial or detrimental for HIV-1 control. In particular, it remains to be determined whether monocytes and macrophages in HIV-1 controllers more efficiently prime NK cells compared to other PLWH, or if in the long-term the crosstalk between these innate effectors predominantly drives chronic inflammation (10–13).

The following review by Bernard et al. comprehensively discusses the role of NK cells in HIV-1 control including involvement in antibody dependent and antibody independent functions. NK cells are regarded as the key cell type better primed to exert antibody dependent cellular cytotoxicity (ADCC) activity earlier in infection. Reports suggest that ECs can maintain high levels of potent gp120 specific antibodies with ADCC or antibody dependent NK cell activation (ADNKA) function in the absence of detectable virus (14, 15), suggesting that an antibody-mediated mechanism may contribute to natural viral control. In addition, there is evidence for a role of NK cells in mediating antibody independent functions (16). It remains to be determined whether antibody dependent activities would provide a better marker for functional cure investigations than antibody independent NK cell responses. The authors further caution on the variability of available assays developed to measure ADCC activity and the importance of assay standardization to overcome differences in antibody titers from person to person before this function can be better defined as a key player in HIV-1 control.

The last review by Sugawara et al. addresses the question of animal models that can recapitulate spontaneous immune control of HIV-1, including humanized mice and non-human primates. Notably, studies in animal models present unique opportunities to evaluate innate immune responses in tissues, within days following HIV-1 infection, or to assess the role of specific innate effector cells using *in vivo* depletion experiments, which are all impossible or challenging in humans. Animal models are also instrumental in pre-clinical studies to evaluate approaches to induce protective immune responses similar to those observed in HIV-1 ECs and the authors discuss possible future immunotherapeutic strategies for PLWH based on harnessing trained immunity, and particularly NK cells with adaptive capabilities.

Trained immunity describes a process by which NK cells, dendritic cells and monocyte/macrophages get ‘primed’ by an initial exposure (i.e., infection) that triggers an epigenetic and metabolic reprograming, allowing them to provide an enhanced response to a subsequent exposure to the same or another unrelated agent (17). HIV-1-induced trained immunity is the main topic covered by the Hypothesis and Theory article by Sviridov et al. Low-level expression of viral antigens, and particularly HIV-1 Nef, likely occurs in ART-suppressed PLWH and HIV-1 controllers (18, 19). The authors speculate that in PLWH, HIV-1 Nef primes myeloid cells, rendering them hyper-responsive to subsequent stimuli, including microbial products translocating from the gastrointestinal tract. In this scenario, trained immunity contributes to sustained inflammation and associated co-morbidities in PLWH. This underscores the need to further investigate trained immunity in HIV-1 infection, as very few studies have focused on the impact of this relatively new concept on HIV-1 control or immunopathology.

Overall, the articles presented here provide an important basis and mechanistic view of how innate immune responses may partner with adaptive immune responses to contain the virus in HIV-1 controllers. Some questions remain regarding the contribution of the innate immune response to the high levels of chronic inflammation and the risk of non-AIDS-related conditions in ECs. It is possible that high levels of immune activation from low-level viral replication trigger sustained immune activation and a chronic inflammatory state that would be beneficial in the early stages of HIV-1 infection for disease containment but detrimental in the chronic state. Therefore, initiation of ART could be a possible solution to avert effects of chronic immune activation in specific populations of ECs. Given that it would be challenging to identify EC populations at high risk of comorbidities, further investigations are required to determine whether treatment should be offered to all EC groups to alleviate the risks.

## Author contributions

All authors listed have made a substantial, direct and intellectual contribution to the work, and approved it for publication.
